# Control and enhancement of optical nonlinearities in plasmonic semiconductor nanostructures

**DOI:** 10.1038/s41377-025-01783-4

**Published:** 2025-05-13

**Authors:** Andrea Rossetti, Huatian Hu, Tommaso Venanzi, Adel Bousseksou, Federico De Luca, Thomas Deckert, Valeria Giliberti, Marialilia Pea, Isabelle Sagnes, Gregoire Beaudoin, Paolo Biagioni, Enrico Baù, Stefan A. Maier, Andreas Tittl, Daniele Brida, Raffaele Colombelli, Michele Ortolani, Cristian Ciracì

**Affiliations:** 1 Department of Physics and Materials Science, University of Luxembourg, 162a avenue de la Faïencerie, Luxembourg, L-1511 Luxembourg; 2 Center for Biomolecular Nanotechnologies, Istituto Italiano di Tecnologia, via Barsanti 14, Arnesano, 73010 Italy; 3 Center for Life Nano- and Neuro-Science, Istituto Italiano di Tecnologia, Viale Regina Elena 291, Rome, 00161 Italy; 4 Centre de Nanosciences et de Nanotechnologies, CNRS UMR 9001, Université Paris-Saclay, Palaiseau, 91120 France; 5 Photonics Initiative, Advanced Science Research Center, City University of New York, New York, 10031 NY USA; 6 Istituto di Fotonica e Nanotecnologie, Consiglio Nazionale delle Ricerche, Via del Fosso del Cavaliere 100, Rome, 00133 Italy; 7 Physics Department, Politecnico di Milano, Piazza Leonardo da Vinci 32, Milan, 20133 Italy; 8 Chair in Hybrid Nanosystems, Nano-Institute Munich, Faculty of Physics, Ludwig-Maximilians-Universtität München, Königinstraße 10, München, 80539 Germany; 9 School of Physics and Astronomy, Monash University, Wellington Rd, Clayton VIC, 3800 Australia; 10 The Blackett Laboratory, Department of Physics, Imperial College London, Wellington Rd, London, SW72AZ UK; 11 Dipartimento di Fisica, Sapienza Università di Roma, Piazzale Aldo Moro 2, Rome, 00185 Italy; 12 Present Address: Neurophos Inc., Austin, TX USA

**Keywords:** Nonlinear optics, Nanophotonics and plasmonics, Ultrafast photonics

## Abstract

The efficiency of nanoscale nonlinear elements in photonic integrated circuits is hindered by the physical limits to the nonlinear optical response of dielectrics, which cannot be engineered as it is a fundamental material property. Here, we experimentally demonstrate that ultrafast optical nonlinearities in doped semiconductors can be engineered and can easily exceed those of conventional undoped dielectrics. The electron response of heavily doped semiconductors acquires in fact a hydrodynamic character that introduces nonlocal effects as well as additional nonlinear sources. Our experimental findings are supported by a comprehensive computational analysis based on the hydrodynamic model. In particular, by studying third-harmonic generation from plasmonic nanoantenna arrays made out of heavily n-doped InGaAs with increasing levels of free-carrier density, we discriminate between hydrodynamic and dielectric nonlinearities. Most importantly, we demonstrate that the maximum nonlinear efficiency as well as its spectral location can be engineered by tuning the doping level. Crucially, the maximum efficiency can be increased by almost two orders of magnitude with respect to the classical dielectric nonlinearity. Having employed the common material platform InGaAs/InP that supports integrated waveguides, our findings pave the way for future exploitation of plasmonic nonlinearities in all-semiconductor photonic integrated circuits.

## Introduction

Nonlinear optics has been historically dominated by experimental configurations where interacting optical beams propagate for distances much longer than the involved wavelengths in bulk nonlinear optical crystals^1^, in optical fibers^2^, or in integrated photonic waveguides^3^. More recently, nonlinear metasurfaces, or nanoantenna arrays of subwavelength thickness^4^ have been introduced to eliminate phase-matching constraints^5^, leading in the latter case to the emergence of *nonlinear plasmonics*^6–8^. Plasmonic nanoantennas are often used as a sub-wavelength field concentrator to enhance the interaction between light and nonlinear dielectric systems and molecules^9–11^. Remarkably, it has been shown that the plasmonic nanostructure itself can also provide a sub-diffraction limit source of optical nonlinearity^6^. However, the mechanism at the origin for this phenomenon is still not fully understood and remains unexploited.

One can identify at least two fundamental mechanisms for instantaneous (i.e. faster than an optical cycle) nonlinear optical response of a plasmonic structure. The first is the dielectric nonlinearity of the bulk material which can be enhanced by the local plasmonic field enhancement (nonlinear dielectric susceptibilities *χ*^(2)^, *χ*^(3)^, etc.). The second mechanism is due to the collective motion of free electrons under an external radiation field. Such nonlocal oscillation is ultimately related to the kinetic energy of the free-electron gas and can be modeled by a set of hydrodynamic equations of motion in analogy with a classical fluid. The distinction between these two mechanisms is crucial since there is a physical limit to the maximum dielectric nonlinearity, related to the form of anharmonic potentials^12^ that sets upper bounds for *χ*^(2)^ and *χ*^(3)^. Such limitation however does not apply to free-electron nonlinearities^1^. Therefore, one may ask whether the free-electron nonlinearity is the dominant effect, and if so, whether it can be harnessed to exceed the limited efficiency of conventional dielectric nonlinearity. Even though the response of a perfectly homogeneous electron gas is intrinsically nonlinear, extracting (i.e. coupling to the far-field) and enhancing nonlinear effects requires nanostructuring the free-electron host medium. In principle, the mere existence of a sharp interface between the host medium and a vacuum could suffice^13^, but in practice much stronger electron density gradients are obtained in nanoantennas under electromagnetic excitation.

In noble metals, and especially in nanoantenna systems, it is extremely difficult to separately investigate the dielectric and free-electron contributions to nonlinearity, as they coexist everywhere in the solid. Doped semiconductors, on the other hand, offer the possibility of controlling the carrier density via external doping, or via a field-effect gate^14–16^, thus allowing to tune the free-electron response while keeping the dielectric nonlinearity constant.

In this work, we combine experiments and theoretical modeling to demonstrate that free electron contributions can dramatically enhance the nonlinear optical response of heavily doped semiconductor nanoantennas. By measuring third harmonic generation (THG) from nanoantennas with different free-electron density and comparing the obtained efficiency to hydrodynamic model calculations, we unveil that the nonlocal free-electron interaction is the fundamental mechanism of nonlinear plasmonics in doped semiconductors. The material platform employed for the experiment is InGaAs/InP because of its broad appeal for the future exploitation of plasmonic nonlinearities in all-semiconductor photonic integrated circuits (PICs)^17–19^.

## Results

The fermionic nature of electron–electron interactions manifests itself as an internal pressure in the electron gas that resists the compression induced by an external electromagnetic field. The effects of such pressure are most apparent near the interfaces between the host material and vacuum or other materials, where strong gradients of the carrier concentration and of the electric field occur. Free-electron nonlinearities^8^ are therefore intrinsically nonlocal, in the sense that the induced currents depend not only on the value of the electric field at a given point but also, through their spatial derivatives, on the value of the fields in the surrounding area^20–22^. The many-body nonlinear and nonlocal dynamics of a free-electron fluid under external electric and magnetic fields, **E**(**r**, *t*) and **H**(**r**, *t*), is described by the following equation for the electron *n*(**r**, *t*) and current **J**(**r**, *t*) densities^23–26^:

1 $${m}^{* }\left(\frac{\partial }{\partial t}-\frac{{\bf{J}}}{en}\cdot \nabla +\gamma \right)\frac{{\bf{J}}}{en}=e{\bf{E}}-\frac{{\bf{J}}}{n}\times {\mu }_{0}{\bf{H}}+\nabla \frac{\delta G[n]}{\delta n}$$

where *μ*_0_ is the magnetic permeability of vacuum, *e* is the electron charge, *γ* is the damping rate of free carrier motion and m* is the electron effective mass that accounts for the band structure in a real solid. The last term on the right-hand side contains the gradient of the functional derivative of the free-energy functional *G*[*n*], i.e., the *quantum pressure* in the electron fluid^13,27^, which can also be obtained from the Thomas–Fermi screening^14^. Here we are neglecting the spatial dependence of *n* at the interface with vacuum(spill-out effect), since the structures we will investigate are relatively large (~1 *μ*m). Following a perturbation approach, it is possible to derive all nonlinear source terms, see details in Supplementary Information (SI)^13,14,27,28^. Here, we report the two THG source terms that appear in the propagating-wave solution of Maxwell’s equations and Eq. (1): the third-order dielectric polarization ${{\bf{P}}}_{{\rm{d}}}^{(3)}$, and the hydrodynamic contribution **S**^(3)^ given by the sum of convective and quantum pressure terms:

2a $${{\bf{P}}}_{{\rm{d,}}3\omega }^{(3)}={\varepsilon }_{0}{\chi }^{(3)}({{\bf{E}}}_{\omega }\cdot {{\bf{E}}}_{\omega }){{\bf{E}}}_{\omega }$$

2b $$\begin{array}{lll}{{\bf{S}}}_{3\omega }^{(3)}=\,-\frac{{\omega }^{2}}{{e}^{2}{n}_{0}^{2}}\left[\nabla \cdot {{\bf{P}}}_{\omega }({{\bf{P}}}_{\omega }\nabla \cdot {{\bf{P}}}_{\omega }+{{\bf{P}}}_{\omega }\cdot \nabla {{\bf{P}}}_{\omega })\right.\\\qquad\,\left.+{{\bf{P}}}_{\omega }\cdot {{\bf{P}}}_{\omega }\nabla \nabla \cdot {{\bf{P}}}_{\omega }\right]+\frac{1}{27}\frac{{\beta }^{2}}{{e}^{2}{{n}_{0}}^{2}}\nabla {(\nabla \cdot {{\bf{P}}}_{\omega })}^{3}\end{array}$$

where **E**_*ω*_ and **P**_*ω*_ = − **J**_*ω*_/*iω* are the field and total polarization vectors at the fundamental frequency *ω* and $\beta =\sqrt{\frac{3}{5}}{v}_{{\rm{F}}}$, where *v*_F_ is the Fermi velocity. One immediately sees that an *effective hydrodynamic susceptibility* cannot be rigorously defined: the additional source ${{\bf{S}}}_{3\omega }^{(3)}$ contains the gradient and the divergence of **P**_*ω*_ and therefore it is strictly nonlocal and proportional to $1/{{n}_{0}}^{2}$. Paradoxically, in noble metals, the high concentration of free-carrier leads to weaker nonlinear contributions. One can understand this behavior considering the material volume that contributes to the nonlinearity: in metals, the polarization gradients are sharp and non-vanishing only at the metal/vacuum interface, resulting in small active nonlinear volumes; in doped semiconductors, the polarization inhomogeneities extend significantly towards the bulk of the material, leading to a much larger active nonlinear volume $V={l}^{3} \sim {({v}_{{\rm{F}}}/{\omega }_{{\rm{p}}})}^{3}\propto 1/({n}_{0}^{1/2}{m}^{* 3/2})$, where *ω*_p_ is the plasma frequency^21,29^. Therefore, the small effective masses m* ~ 0.1*m*_*e*_ of electrons in semiconductors and their comparatively low carrier density lead to an increased active volume, and eventually to a higher global efficiency, of nonlinearity if compared to noble metals. In the absence of spatial variations of **P**_*ω*_, the nonlinear source term of Eq. (2) vanishes and the hydrodynamic model reduces to the Drude model with the permittivity ${\varepsilon }_{{\rm{r}}}={\varepsilon }_{\infty }\left(1-\frac{{\omega }_{{\rm{p}}}^{2}}{{\omega }^{2}+i\gamma \omega }\right)$, with the plasma frequency ${\omega }_{{\rm{p}}}=\sqrt{{n}_{0}{e}^{2}/{\varepsilon }_{0}{\varepsilon }_{\infty }{m}^{* }}$, where *ε*_*∞*_ is the infinity dielectric constant of the semiconductor (see SI). The plasma wavelength ${\lambda }_{{\rm{p}}}=\frac{2\pi c}{{\omega }_{{\rm{p}}}}$ will be used throughout this article, with *c* being the speed of light in vacuum.

Heavily doped semiconductors display a broad range of *λ*_p_ values in the infrared (IR) spectrum depending on *ε*_*∞*_, m* and *n*_0_. In practice, if one restricts to small m* materials compatible with modern PIC nanofabrication processes, the choice reduces to Ge or SiGe grown on Si (group-IV)^30^, In_0.53_Ga_0.47_As grown on InP (InGaAs/InP), and InAs_0.9_Sb_0.1_ grown on GaSb (III-V)^31^. All these material systems allow for a limited dopant incorporation which in practice bounds *λ*_p_ > 5 *μ*m, i.e., to the mid-IR. In this work, we have used electron-doped InGaAs/InP with various dopant densities (m* = 0.041*m*_*e*_, *ε*_*∞*_ = 12, *n*_0_ ≤ 1 × 10^19^ cm^−3^), and utilized fundamental fields (FF) with center wavelength *λ*_FF_ ranging from 12 to 6 *μ*m to drive the nonlinearity. InP has a bandgap energy around 1.35 eV, i.e. absorption edge around 950 nm, which makes it perfectly suitable for infrared applications including supporting PICs (index of refraction 3.1 in the mid-IR).

**Fig. 1:**
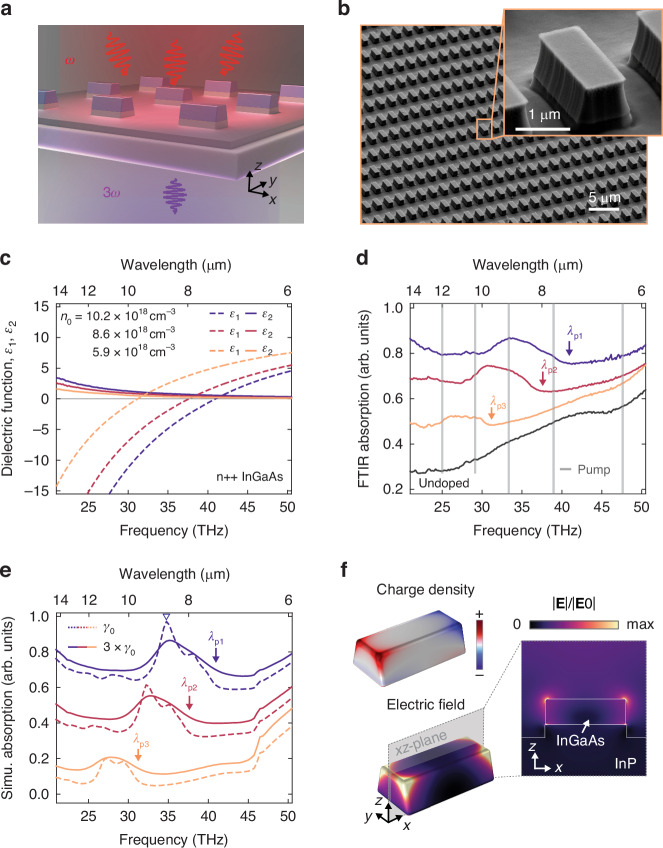
Plasmonic antenna arrays. **a** Schematic illustration of the InGaAs plasmonic antenna on dielectric InP, and the nonlinear experiment producing third harmonic radiations. **b** Scanning electron microscope image of the antennas, featuring length, width and thickness approximately equal to 2.2 *μ*m, 0.8 *μ*m and 0.8 *μ*m, respectively. **c** Real and imaginary parts of the dielectric function of n-doped InGaAs thin layers used to fabricate the plasmonic nanoantenna. Experimental (**d**), and theoretical (**e**) absorption infrared spectra of the antenna arrays for four different doping: undoped (black) to maximum doping (blue). The spectra are shifted vertically for clarity in both plots. Their corresponding plasma wavelengths were indicated by arrows labeled *λ*_p1,2,3_. The gray vertical lines indicate the fundamental field wavelengths used in the nonlinear experiments. The dashed and solid lines in simulation (**e**) correspond to the results with the original *γ*_0_ from Drude-model fitting of pristine InGaAs on InP, and an effective decay 3*γ*_0_ which broadens the linewidth and match the experimental absorption, respectively. The additional damping might originate from the geometry imperfections and from the RIE processes, for example due to the inhomogeneous free carrier depletion at the different antenna surfaces. **f** The field and induced charge distributions of the main plasmonic resonance marked in (**e**)

Three InGaAs films with different doping levels were grown on undoped InP substrates (see Methods). The InGaAs permittivity *ε*_r_(*ω*) = *ε*_1_ + *iε*_2_ was retrieved from the absolute thick-film (~3 *μ*m) reflectivity measured by Fourier-transform infrared spectroscopy (FTIR) using the Kramers-Kroenig relations (Fig. 1c). A Drude fit to *ε*_r_(*ω*) provides *n*_0_ = 1.02 × 10^19^, 8.6 × 10^18^ and 5.9 × 10^18^ cm^−3^, for samples 1, 2, and 3 respectively, and a doping-independent scattering rate *γ*_0_ = 8.9 THz. *λ*_p_ = 7.33, 7.97, and 9.62 *μ*m were directly obtained as the zero-crossing point of *ε*_1_ in Fig. 1c^32^. Photo-excitation of electron–hole plasmas in undoped semiconductors is an alternative method to study even higher free-electron density plasmas, but the temporal^33^ and spatial^34^ dynamics are more complex and may obscure the hydrodynamic behavior of free electrons. The InGaAs nanoantennas, consisting of periodically arranged rectangular rods with slightly trapezoidal cross-sections, have been fabricated by etching the excess InGaAs down to the InP substrate by deep reactive ion etching (RIE) through a mask produced by electron-beam lithography, as shown in Fig. 1b. As anticipated in the introduction, the choice to conduct the experiment using rectangular antennas instead of a thin film, which is also feasible (see SI), was made to enhance the differences between dielectric nonlinear susceptibility *χ*^(3)^ and the hydrodynamic nonlinearity due to the much larger volume with significant polarization gradients.

The antenna arrays have been characterized by FTIR transmission/reflection microscopy: for light polarized along the antenna axis, they display localized surface plasmon resonances (LSPR) around 8.7, 9.4 and 10.9 *μ*m for the different doping levels, as shown in Fig. 1d. These resonances could be well reproduced by numerical full-wave electromagnetic simulations carried out using the finite-element method, as shown in Fig. 1e (see Methods and SI). It is worth noting that, apart from using the decay rate *γ*_0_ from the Drude fit, we also introduce an “effective" decay of 3*γ*_0_ to account for the overall damping that dissipates energy from the system while not differentiating the radiative and nonradiative channels. Figure 1f displays the induced charge density and electric field of the LSPR, revealing a high-order plasmonic behavior^35^. The LSPRs energies are insensitive to the geometric dimensions of the antennas and pinned to be close to *λ*_p_ since the antenna length is shorter than the plasma wavelengths of the InGaAs layer, which is a typical behavior of plasmonic resonances^36^. We investigate antennas of different sizes (FTIR spectra shown in SI) and we observe that their LSPRs do not shift in energy even if their size changes.

**Fig. 2:**
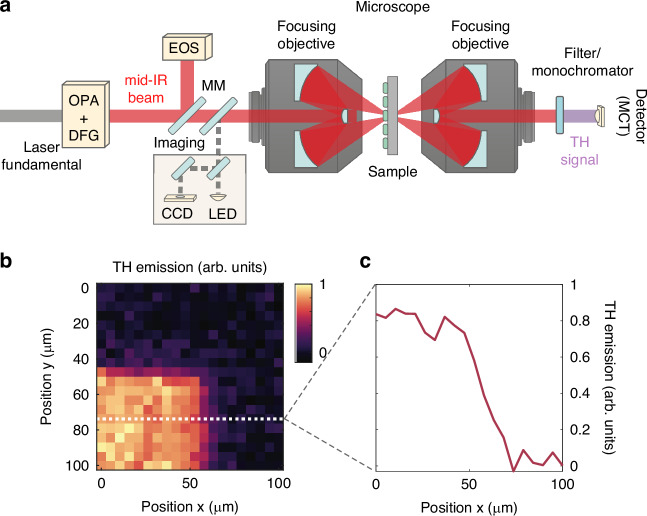
Mid-infrared THG from antenna arrays. **a** Schematic representation of the experimental setup used for the THG experiment. The fundamental beam out of a Yb:KGW laser amplifier is used to drive a tunable mid-IR source, based on difference frequency generation (DFG) between a noncollinear optical parametric amplifier (OPA) and the laser fundamental. Using a mirror on a magnetic mount (MM) the generated mid-IR transients can be characterized by means of electro-optic sampling (EOS). The mid-IR pulses are coupled into the InGaAs nanoantennas through a reflective microscope and filtered after the interaction with the sample. The emitted third-harmonic signal is collected and measured by an MCT detector. **b** Map of third harmonic emission from one of the nanoantenna arrays used in this experiment. **c** Profile of third harmonic emission as the beam position is scanned across the array edge (dotted lines in **b**)

The hydrodynamic nonlinear response shows different regimes depending on the ratio between *λ*_FF_ that drives the emission and *λ*_p_, which in turn depends on *n*_0_. To generate different *λ*_FF_ we have employed a pulsed mid-IR laser source, tunable between 5 and 15 *μ*m, and we have tightly focused the beam at the diffraction limit (Fig. 2a, see Methods). The antenna arrays were mounted on a three-dimensional micro-positioner, so as to obtain a two-dimensional map in the focal plane of the third-harmonic emission^37^. The strong THG from the antennas is in large contrast to the weak contribution from the substrate as depicted in Fig. 2b, c (*χ*^(3)^ = 1.4 × 10^6^ pm^2^/V^2^ in InGaAs, *χ*^(3)^ = 1.0 × 10^6^ pm^2^/V^2^ in InP^11^).

**Fig. 3:**
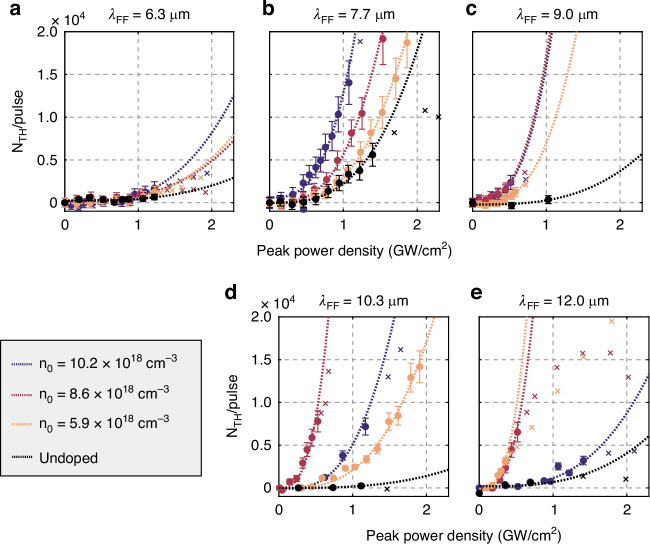
Power dependences of the mid-infrared THG. **a**–**e** Fluence dependence of the THG at different doping levels (color coded) and different fundamental field wavelengths *λ*_FF_ (different panels). A cubic fit model *N*_TH_ = *a* + *η*_*THG*_*x*^3^ extracts the nonlinear coefficient, omitting data points (*x*) within a saturation regime

THG emission itself is confirmed by measuring a spectrum of the emitted radiation with a dispersive spectrometer (compare SI) while ensuring that other orders of nonlinearity (mainly second) are filtered out. The intensity of the THG allows to calculate the number of third harmonic photons *N*_TH_ emitted per pulse by the antenna array as a function of pump peak power density (Fig. 3a–e). The coefficient of the cubic fits defines the nonlinear efficiency of the THG process *η*_*THG*_ for each pair of *λ*_FF_ and *n*_0_ values. Above a certain threshold, we observe a deviation from the expected cubic behavior due to heating^38^ and/or free electron current saturation effects in high driving fields^37^, and the corresponding data points are omitted in the fitting. The values of *λ*_FF_ = 6.3 *μ*m, 7.7 *μ*m, 9.0 *μ*m, 10.3 *μ*m, and 12.0 *μ*m are above, close to, or below *λ*_p_ = 9.62, 7.97, and 7.33 *μ*m for the three samples. The undoped reference nanoantenna sample, which eliminates free-electrons contributions of nonlinearity, showed very weak THG for all *λ*_FF_.

## Discussion

**Fig. 4:**
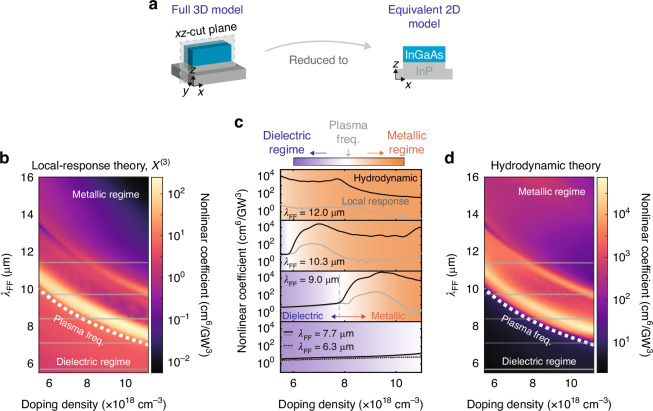
Numerical analysis of the equivalent 2D system. **a** Schematic of the equivalent 2D model. Maps of nonlinear coefficient of the single antenna with different fundamental field (FF) wavelengths and doping density based on (**b**), local model with only *χ*^(3)^, or (**d**), nonlocal hydrodynamic model with both *χ*^(3)^ and hydrodynamic sources. White dotted lines indicate the screened plasma wavelength on different doping densities which infers the condition *Re*(*ε*) = 0. The gray horizontal lines indicate the fundamental field wavelengths used in the experiments but with a 0.6 *μ*m blueshift due to the correction between 2D and 3D models as discussed in the SI. (**c**) indicates the specific nonlinear coefficients under the experimental configurations based on the local-response (gray lines) or hydrodynamic (black lines) model. In the dielectric regime, theoretical results of *λ*_FF_ = 6.3 *μ*m (dashed lines) are comparable with *λ*_FF_ = 7.7 *μ*m. The colormap indicates different regimes

To reveal the mechanism at the origin of the free-carrier-density dependent THG, we have numerically solved Eq. (1) together with the wave equation, following a perturbative approach using a finite-element method (see SI), with *n*_0_ and *λ*_FF_ as free parameters. Because of the rapid variations of the fields at the semiconductor surface introduced by the hydrodynamic terms, it is computationally very challenging to perform full three-dimensional (3D) calculations of the antenna system^39^. Here we used the two-dimensional (2D) equivalent model of Fig. 4a to simulate the single antenna. The 2D model reproduces the main linear spectral characteristics of the 3D system in Fig. 1d as long as a systematic shift of approximately Δ*λ* ≃ −0.6 *μ*m is taken into account (see SI). More importantly, the absorption spectra of the 3D antenna array align well with that of the 3D single antenna (SI), revealing a negligible inter-antenna coupling. This is due to the large gap between every two antennas. This fact validates our strategy of independent-antenna approximation with which the nonlinear coefficient could be scaled by the number of antennas involved when compared with experiments. The numerical nonlinear efficiencies of a single antenna are summarized in Fig. 4b–d, where we show color maps of the nonlinear coefficient ${\eta }_{THG}=\frac{{N}_{{\rm{TH}}}}{{I}_{{\rm{FF}}}^{3}}$ as a function of *λ*_FF_ and *n*_0_, with *N*_TH_ being the THG photon count per pulse and *I*_FF_ the fundamental field power density in GW/cm^2^.

In Fig. 4b we considered a local-response theory with the dielectric susceptibility *χ*^(3)^ as the only source of nonlinearity (Eq. (2a) where ${{\bf{S}}}_{3\omega }^{(3)}$ is artificially set to zero). Single particle nonlinearities due to non-parabolicity are of the same order of dielectric nonlinearities in heavily doped semiconductors^40^, therefore much weaker than hydrodynamic nonlinearities as we show here. The plasmonic field enhancement of the nanoantennas is included in the calculation as a Drude term. In Fig. 4d both nonlinear contributions of Eq. 2 and (2b) were included (see also pure hydrodynamic contributions in SI). The strong dependence of *η*_*THG*_ on *n*_0_ is markedly different at each *λ*_FF_ as we highlight in Fig. 4c, where we plot a few selected horizontal cuts of the color maps.

We can identify three different regimes: i) the *dielectric regime*, when *λ*_FF_ < *λ*_p_, i.e., below the white dotted line in Fig. 4b, d and in the bottom panel (*λ*_FF_ = 6.3 and 7.7 *μ*m) of Fig. 4c; ii) the *plasmonic resonance regime*, when *λ*_FF_ ≃ *λ*_LSPR_, i. e., the bright regions just above the white dotted line in Fig. 4b, d and in the *λ*_FF_ = 9.0 and 10.3 *μ*m panels in Fig. 4c); iii) the metallic regime when *λ*_FF_ > *λ*_p_ as in the upper parts of Fig. 4b, d and in the top panel (*λ*_FF_ = 12.0 *μ*m) of Fig. 4c.

Considering the local response (gray curves in Fig. 4c) we observe an enhancement of *η*_*THG*_ above the dielectric-*χ*^(3)^ level of 10^1^ cm^6^/ GW^3^ only in the plasmonic resonance regime (broad peaks at *n*_0_ ~ 7 × 10^18^ cm^−3^ for *λ*_FF_ = 10.3 *μ*m and *n*_0_ ~ 9.5 × 10^18^ cm^−3^ for *λ*_FF_ = 9.0 *μ*m). This is due to the increase of the linear extinction cross-section of the antennas at the LSPR, which effectively increases the polarization field within the material. The peak value of *η*_*THG*_ is in the range 10^2^ cm^6^/GW^3^, 20 times higher than the dielectric-*χ*^(3)^ baseline level. In the metallic regime at *λ*_FF_ = 12.0 *μ*m, *η*_*THG*_ drops to zero for high *n*_0_, because there is very small field penetration in the material.

Considering the hydrodynamic case (black curves in Fig. 4c), *η*_*THG*_ is generally much higher than in the local case, apart from the dielectric regime (violet background in Fig. 4c). In the plasmonic resonance regime, *η*_*THG*_ shows a broad enhancement at similar *n*_0_ values as for the local theory, and the magnitude of the enhancement is 200 times stronger (in total, almost 5000 times stronger than the dielectric-*χ*^(3)^ baseline level). This cannot be accounted for by the pump extinction enhancement, which affects both source terms equally, therefore it must be due to the hydrodynamic nonlinearity of Eq. (2b). Contrarily to the local-response theory, the nonlinear coefficient enhancement is still visible in the metallic regime, where plasmon fields at the semiconductor surface still exist even out of resonance: the very small field penetration in the bulk does not impact on the hydrodynamic nonlinearity, which originates close to the antenna surface, where gradients are strongest. For *λ*_FF_ = 12.0 *μ*m, *η*_*THG*_ is nonzero for high *n*_0_ and it is especially strong for decreasing *n*_0_, up to 10^4^ cm^6^/GW^3^.

We now compare the experimental data with the numerical calculations performed with an “effective" decay *γ* = 3*γ*_0_ to account for the overall damping due to the imperfection that broadens the resonances as observed with linear optical characterization. To compare the numerical THG efficiency with the experimental one, we have estimated the beam width at full-width-half-maximum to be ~80 *μ*m, which implies that ~640 antennas contributed to the measured THG.

**Fig. 5:**
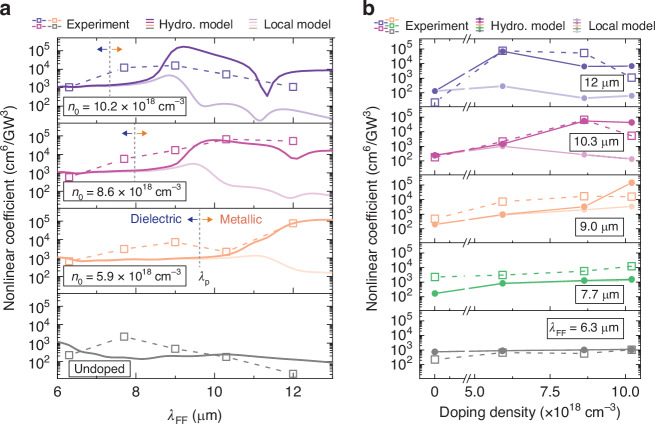
Comparison of experiments with different theories. **a** Nonlinear coefficients as a function of the fundamental field wavelength *λ*_FF_ of different doping densities *n*_0_. Experimental data (markers with dashed lines) was compared with theoretical results from hydrodynamic theory (darker solid lines), and classical local-response model with only a dielectric *χ*^(3)^ (lighter solid lines). Metallic and dielectric regimes are separated by a dashed line at plasma wavelength *λ*_p_. **b** Nonlinear coefficients as a function of doping densities, excited with different *λ*_FF_ acquired from the experiments (markers), hydrodynamic theory (darker solid lines), and classical local-response model (lighter solid lines). Numerical data were obtained by properly normalizing 2D calculations and considering a broadened damping of 3*γ*_0_ (see SI)

The *η*_*THG*_ retrieved from the cubic fits in Fig. 3a–e are summarized in Fig. 5a as a function of *λ*_FF_. At high doping densities and long wavelengths *λ*_*FF*_ (*metallic regime*), Fig. 5a shows that the nonlinear coefficient predicted by hydrodynamic theory matches the experimental data, in stark contrast with the results of the local-response theory. In this regime, the nonlinear coefficients predicted by the hydrodynamic theory, proved by the experiments, have a two-orders of magnitude enhancement (~10^5^ cm^6^/GW^3^) compared with the local-response model (~10^3^ cm^6^/GW^3^). For the undoped case (lower panel of Fig. 5a), the two theories predict the same results due to the lack of free electrons and the absence of free-electron nonlinearity (*dielectric regime*). The theories predict low and spectrally flat optical nonlinearity, matching the experimental results.

Figure 5b represents the nonlinear THG coefficient as a function of doping density *n*_0_, to compare the experimental results with Fig. 4. At the shortest fundamental field wavelength *λ*_*FF*_ = 6 *μ*m, the two theories overlap and predict low nonlinearity. In contrast, at the highest doping and long wavelength (*metallic regime*), the high THG coefficients can be explained only within the hydrodynamic theory. The three experimental conditions that have provided the highest *η*_*THG*_ are *λ*_FF_ = 12.0 *μ*m (blue squares) and *n*_0_ = 5.9 and 8.6 × 10^18^ cm^−3^, and *λ*_FF_ = 10.3 *μ*m (violet squares) and *n*_0_ = 8.6 × 10^18^ cm^−3^. Remarkably the hydrodynamic model and the experimental nonlinear coefficients are of the same order of magnitude (~10^5^ cm^6^/GW^3^). In summary, the experiment agrees well with the main hydrodynamic model predictions in Fig. 5, where efficiencies in the metallic regime (long *λ*_FF_) are generally much higher than in the dielectric regime (short *λ*_FF_), while the local-response model in Fig. 5f predicts exactly the opposite behavior.

## Conclusion

The combination of theory and experiments allows us to demonstrate that the fundamental origin for THG in optical nanoantennas made of heavily doped semiconductors is the nonlinear collective behavior of free electrons, described within a hydrodynamic formalism, as opposed to the conventional dielectric nonlinearity due to crystal lattice anharmonicity and bound electrons. which is described by a local susceptibility *χ*^(3)^ independent of the doping level. The experiments show that the efficiency of THG could be up to two orders of magnitude larger than the dielectric one in InGaAs.

We can thus speculate then that free electrons might also be the predominant source of nonlinearity in all possible plasmonic systems and therefore might be relevant to a wide range of nonlinear experiments that involve gold nanoantenna arrays^41^. In this context, shorter length scales, stronger fields and higher energy loss might require further developments even beyond the hydrodynamic description presented, with interesting perspective of understanding collective oscillations in free electron gasses^22^. In addition, the employed semiconductor material platform (InGaAs/InP) is currently under scrutiny to realize photonic integrated circuits in the mid-IR, featuring all-semiconductor waveguides and resonators^17–19^. Plasmonic effects, introduced by selectively doping specific volumes, could provide such photonic integrated circuits with tailored giant nonlinear coefficients. If realized, this new type of tunable nonlinear photonic circuit holds promise for nonlinear signal processing.

Finally, our study underscores the importance of a holistic approach in the design of optical nanoantennas. The local theory allows for the identification of the nonlinear source distribution with the local optical pump intensity patterns. Instead, to quantify the hydrodynamic nonlinearity the full equations of motion of the electron fluid in an external optical field must be solved for each specific geometry. In summary, the nonlocal hydrodynamic response adds a layer of complexity to nonlinear plasmonic device design, but it also unlocks a richer landscape of opportunities.

## Materials and methods

### Growth and antenna fabrication

The InGaAs thin films were grown by MOCVD (Metal Organic Chemical Vapor Deposition) on InP substrates. The films were doped with Si leading to a n-doping of the material. The thickness of the InGaAs film was about 3 *μ*m. The doping levels of the thin films were calculated by measuring the reflectance by means of Fourier-transform infrared spectroscopy (FTIR) and by performing a Drude fit. The antenna arrays were fabricated by lithography and etching the InGaAs film, after thinning down the InGaAs epi-layer to 800 nm with wet chemical etching.

### Linear characterization

The antenna arrays were investigated by micro-FTIR spectroscopy to measure their plasmonic resonances. The measurements were carried out with a commercial Bruker IFS-66V Michelson interferometer coupled to an infrared microscope (*Hyperion*). The objective was reflective cassegrain-type with a numerical aperture (NA) of 0.4 and a magnification of 15x. The detector is a liquid nitrogen-cooled Mercury Cadmium Telluride (MCT). The FTIR measurements of the antenna arrays were performed both in reflection (*R*) and in transmission (*T*), and the absorption coefficients shown in Fig 1d were calculated as 1−*R*−*T*.

### Nonlinear characterization

Our tunable mid-IR source is based on a Yb:KGW laser amplifier, emitting 100 *μ*J pulses with 1030 nm central wavelength and operating at a repetition rate of 100 kHz. Fundamental wavelength (FW) pulses with energy of 50 *μ*J drive a noncollinear optical parametric amplifier (NOPA), delivering broadband near-infrared pulses tunable in the range between 1050 and 1400 nm and 1 *μ*J pulse energy. The output of the NOPA and the remaining 50 *μ*J of laser FW are collinearly focused onto a 1.2-mm-thick GaSe crystal, where p-polarized mid-IR pulses (with pulse energy up to 100 nJ) are generated by means of difference frequency generation (DFG) in a type-II configuration. The spectrum of the mid-IR pulses can be tuned by a suitable selection of the NOPA output central wavelength along with careful adjustments of the phase-matching conditions. The resulting mid-IR transients are characterized by means of electro-optic sampling, yielding for all the excitation pulses used in this work a temporal duration of 400 fs, a bandwidth of 1.5 THz and peak electric fields up to 10 MV/cm. The mid-IR pulses are coupled into the InGaAs antennas using a confocal microscope operating in transmission geometry and based on gold-coated dispersionless Cassegrain reflective objectives with 0.5 numerical aperture (NA).

The microscope can also be used in reflective geometry to image the sample and locate the antenna arrays. The emitted third harmonic radiation is measured using a liquid-nitrogen-cooled MCT detector and lock-in readout. In order to filter the fundamental mid-IR pulse from the third-harmonic emission, we have used a 5mm thick sapphire window, which acts as a short pass filter with transmission edge at 5 *μ*m. In order to filter spurious second harmonic emission from the substrate we have employed either crystalline filters or a monochromator, depending on the excitation wavelength. The monochromator has also been used to record the spectrum of the third-harmonic emission from the antennas.

To calculate the number of TH photons from the voltage signal at the MCT detector, we used the following calibration procedure. We accounted for the wavelength sensitivity of the photovoltaic MCT detector and with the loss factors due to lenses and glass filters. When we performed measurements with the monochromator, we have also considered the spectral efficiency of the grating and the finite bandwidth of the monochromator output. We have corrected this by comparing the third harmonic signal both with the monochromator and using glass filters at the same wavelength. To transform the detector output voltage into a number of photons emitted, we have then measured the fundamental beam (at *λ*_*FF*_=12.0*μ*m) both with a thermal power meter and with the MCT detector. The power value is then converted into the number of photons N emitted per pulse using the relation *P* = *E*_*pulse*_*f* = *N*_*photons*_*hνf* where *E*_*pulse*_ is the pulse energy, h is the Planck constant, *ν* the central frequency of third harmonic emission and f is the repetition rate of the laser.

### Simulations

We used the finite-element method (COMSOL Multiphysics) to solve the differential equation system formed by the free-electrons equation and the electromagnetic wave equation in the frequency domain. The customized coupled equations were implemented using proper weak-form expressions. Overall, three steps, where each step solving for each harmonic (*ω*, 2*ω*, 3*ω*), were used to take both cascaded and direct THG into account, see details in SI.

### Online content

Any methods, additional references, Nature Research reporting summaries, source data, extended data, supplementary information, acknowledgments, peer review information; details of author contributions and competing interests; and statements of data and code availability are available at 10.1038/s41377-025-01783-4.

## Supplementary information

Control and enhancement of optical nonlinearities in plasmonic semiconductor nanostructures

## Data Availability

Source data are available for this paper. All other data that support the plots within this paper and other findings of this study are available from the corresponding author upon reasonable request.

## Code availability

The COMSOL models are available from the corresponding author upon reasonable request.
