# Supplementary material for: Control and enhancement of optical nonlinearities in plasmonic semiconductor nanostructures
Source: Light Sci Appl. 2025 May 13;14:192. doi: 10.1038/s41377-025-01783-4 (PMC12075597; doi:10.1038/s41377-025-01783-4)
Supplement: Supplementary file 1 — Control and enhancement of optical nonlinearities in plasmonic semiconductor nanostructures [file 41377_2025_1783_MOESM1_ESM.pdf]

Supplementary Information

Control and enhancement of optical nonlinearities in plasmonic semiconductor nanostructures

Andrea Rossetti[1†], Huatian Hu[2†], Tommaso Venanzi[3†], Adel Bousseksou[4], Federico De Luca[5], Thomas Deckert[1], Valeria Giliberti[3], Marialilia Pea[6], Isabelle Sagnes[4], Gregoire Beaudoin[4], Paolo Biagioni[7], Enrico Baù[8], Stefan A. Maier[9,10], Andreas Tittl[8], Daniele Brida[1], Raffaele Colombelli[4], Michele Ortolani[3,6,11*], Cristian Ciracì[2,12*]

[1]Department of Materials Science, University of Luxembourg, 162a avenue de la Faïencerie, Luxembourg, L-1511, Luxembourg.
[2]Center for Biomolecular Nanotechnologies, Istituto Italiano di Tecnologia, via Barsanti 14,Arnesano, 73010, Italy.
[3]Center for Life Nano- and Neuro-Science, Istituto Italiano di Tecnologia, Viale Regina Elena 291,Rome, 00161, Italy.
[4]Centre de Nanosciences et de Nanotechnologies, CNRS UMR 9001, Université Paris-Saclay, Palaiseau, 91120, France.
[5]Photonics Initiative, Advanced Science Research Center, City University of New York, New York, 10031, NY, USA.
[6]Istituto di Fotonica e Nanotecnologie, Consiglio Nazionale delle Ricerche, Via del Fosso del Cavaliere 100, Rome, 00133, Italy.
[7]Physics Department, Politecnico di Milano, Piazza Leonardo da Vinci 32, Milan, 20133, Italy.
[8]Chair in Hybrid Nanosystems, Nano-Institute Munich, Faculty of Physics, Ludwig-Maximilians-Universtität München, Königinstraße 10, München, 80539, Germany.
[9]School of Physics and Astronomy, Monash University, Wellington Rd, Clayton VIC, 3800, Australia.
[10]The Blackett Laboratory, Department of Physics, Imperial College London, Wellington Rd, London, SW72AZ, United Kingdom.

[11]Dipartimento di Fisica, Sapienza Università di Roma, Piazzale Aldo Moro 2, Rome, 00185, Italy.
[12]Neurophos Inc., Austin TX, United States (Current affiliation).

*Corresponding author(s). E-mail(s): michele.ortolani@uniroma1.it; cristian@neurophos.com;
†These authors contributed equally to this work.

The hydrodynamic equations

For convenience, let us rewrite here Eq. (1) from the main article describing the dynamics of the electron fluid under the influence of external electric and magnetic fields, $\mathbf{E}(\mathbf{r}, t)$ and $\mathbf{H}(\mathbf{r}, t)$:

$$m^* \left(\frac{\partial}{\partial t} - \frac{\mathbf{J}}{en} \cdot \nabla + \gamma \right) \frac{\mathbf{J}}{en} = e\mathbf{E} - \frac{\mathbf{J}}{n} \times \mu_0 \mathbf{H} + \nabla \frac{\delta G[n]}{\delta n} \tag{S1}$$

where μ_0 is the magnetic permeability of vacuum, γ is the damping rate of free carrier motion and m^* has replaced m in a real solid.

Within the limit of a single orbital, Eq. (S1) is exact if the kinetic energy of the system is described through the von Weizsäcker functional $\frac{\delta T_{\mathrm{vW}}}{\delta n} = -\frac{1}{2} c_{\mathrm{vW}} \frac{\nabla^2 \sqrt{n}}{\sqrt{n}}$ [1, 2], with $c_{\mathrm{vW}} = \frac{\hbar^2}{m^*}$. Beyond the single particle case, the validity of Eq. (S1) can be recovered in the limit of many electrons by including the Thomas-Fermi correction, $\frac{\delta T_{\mathrm{TF}}}{\delta n} = c_{\mathrm{TF}} \frac{5}{3} n^{\frac{2}{3}}$, with $c_{\mathrm{TF}} = \frac{\hbar^2}{m^*} \frac{3}{10} (3\pi^2)^{2/3}$, to the kinetic energy functional such that $\frac{\delta G[n]}{\delta n} \simeq \frac{\delta T_{\mathrm{TF}}[n]}{\delta n} + \frac{\delta T_{\mathrm{vW}}[n]}{\delta n}$. The term $\frac{\delta T_{\mathrm{vW}}[n]}{\delta n}$ is mostly relevant when variation of the equilibrium densities are considered, and since we are considering relatively large systems, for sake fo simplicity, we neglect this therm. Throughout this article, then, we consider the following approximation of the kinetic energy functional, $\frac{\delta G[n]}{\delta n} \simeq \frac{\delta T_{\mathrm{TF}}[n]}{\delta n}$.

Following a perturbative approach, it is possible to write Eq. (S1) as:

$$\ddot{\mathbf{P}} + \gamma \dot{\mathbf{P}} = \frac{n_0 e^2}{m} \mathbf{E} + \beta^2 \nabla (\nabla \cdot \mathbf{P}) + \mathbf{S}_{\mathrm{NL}}^{(2)} + \mathbf{S}_{\mathrm{NL}}^{(3)} \tag{S2}$$

where time derivatives are now expressed in dot notation and $\dot{\mathbf{P}} = \mathbf{J}$, $\beta^2 = \frac{10}{9} \frac{c_{\mathrm{TF}}}{m} n_0^{2/3}$, with n_0 being the constant equilibrium charge density, and the second- and third-order nonlinear sources, $\mathbf{S}_{\mathrm{NL}}^{(2)}$ and $\mathbf{S}_{\mathrm{NL}}^{(3)}$ are:

$$\begin{aligned} \mathbf{S}_{\mathrm{NL}}^{(2)} = {} & \frac{e}{m} \mathbf{E} \nabla \cdot \mathbf{P} - \frac{e\mu_0}{m} \dot{\mathbf{P}} \times \mathbf{H} + \frac{1}{en_0} (\dot{\mathbf{P}} \nabla \cdot \dot{\mathbf{P}} + \dot{\mathbf{P}} \cdot \nabla \dot{\mathbf{P}}) \\ & + \frac{1}{3} \frac{\beta^2}{en_0} \nabla (\nabla \cdot \mathbf{P})^2 \end{aligned} \tag{S3a}$$

$$\mathbf{S}_{\rm NL}^{(3)} = -\frac{1}{e^2 n_0^2}\left[\nabla\cdot\mathbf{P}(\dot{\mathbf{P}}\nabla\cdot\dot{\mathbf{P}}+\dot{\mathbf{P}}\cdot\nabla\dot{\mathbf{P}})+\dot{\mathbf{P}}\cdot\dot{\mathbf{P}}\nabla\nabla\cdot\mathbf{P}\right] - \frac{1}{27}\frac{\beta^2}{e^2 n_0^2}\nabla(\nabla\cdot\mathbf{P})^3 \tag{S3b}$$

As it can be noted, second-order terms include both local and nonlocal (those containing the gradient or divergence of $\mathbf{P}$) terms, i.e., Coulomb, Lorentz, convective and quantum pressure contributions in that order in Eq. S3a, while third-order terms in Eq. S3b only include nonlocal terms, i.e., convective and pressure contributions.

In order to describe the above mentioned process, let us assume time-harmonic dependence of the fields, i.e. $\mathbf{F}(\mathbf{r},t) = \sum_j \mathbf{F}_j(\mathbf{r})e^{-i\omega_j t}$, with $\mathbf{F} = \mathbf{E}$, $\mathbf{H}$, or $\mathbf{P}$.

Eq. (S2) with Eqs. (S3) and Maxwell's equations can be rewritten as a set of equations for each harmonic ω_j as follows [3]:

$$\nabla\times\nabla\times\mathbf{E}_j - \varepsilon_\infty\frac{\omega_j^2}{c^2}\mathbf{E}_j - \omega_1^2\mu_0(\mathbf{P}_j + \mathbf{P}_{{\rm b},\omega_j}) = 0 \tag{S4a}$$

$$\beta'^2\nabla(\nabla\cdot\mathbf{P}_j) + (\omega_j^2 + i\gamma\omega_j)\mathbf{P}_j = -\frac{n_0 e^2}{m^*}\mathbf{E}_j + \mathbf{S}_{\omega_j} \tag{S4b}$$

where $\mathbf{P}_j = -\mathbf{J}_j/i\omega_j$, and $\beta'^2 = \frac{9}{5}\beta^2$. The coefficient $\frac{9}{5}$ is a correction factor introduced to match the hydrodynamic linear response k^2-dependence to the Lindhard function expansion [4].

In writing these equations we have also considered dielectric local contributions both linear, through the local relative permittivity ε_∞, and nonlinear, through the nonlinear polarization $\mathbf{P}_{{\rm b},\omega_j}$. Coupling between different harmonics occurs through the nonlinear source terms $\mathbf{P}_{{\rm b},\omega_j}$ and $\mathbf{S}_{\omega_j}$. For simplicity, we assume that the pump field is not affected by the nonlinear process (undepleted pump approximation), i.e. $\mathbf{P}_{\omega_1}^{\rm NL} = \mathbf{S}_{\omega_1} = 0$, as we expect harmonic signals to be several orders of magnitude smaller than the pump fields. The system of Eqs. (S4) reduces then to three sets of one-way coupled equations, one for the fundamental ($j = 1$), one for the second-harmonic frequency (j = 2) and one for the third-harmonic frequency ($j = 3$).

The term that takes into account crystal lattice nonlinearities can be described using a bulk third-order susceptibility $\chi^{(3)}$ as:

$$\mathbf{P}_{\omega_3}^{\rm NL} = \varepsilon_0\chi^{(3)}(\mathbf{E}_1\cdot\mathbf{E}_1)\mathbf{E}_1 \tag{S5}$$

In writing this equation we have assumed a centrosymmetric material, which also implies $\chi^{(2)} = 0$. The nonlinear source terms due to free charges are:

$$\mathbf{S}_{\omega_2} = -\frac{e}{m}(\mathbf{E}_1\nabla\cdot\mathbf{P}_1) - i\frac{e\mu_0}{m}\omega_1\mathbf{P}_1\times\mathbf{H}_1 + +\frac{\omega_1^2}{en_0}(\mathbf{P}_1\nabla\cdot\mathbf{P}_1 + \mathbf{P}_1\cdot\nabla\mathbf{P}_1) + -\frac{2}{3}\frac{\beta^2}{en_0}(\nabla\cdot\mathbf{P}_1\nabla\nabla\cdot\mathbf{P}_1) \tag{S6}$$

for the SHG and $\mathbf{S}_{\omega_3} = \mathbf{S}^{(2)}_{\omega_3} + \mathbf{S}^{(3)}_{\omega_3}$ for the THG, with :

$$\begin{aligned}\mathbf{S}^{(2)}_{\omega_3} = &-\frac{e}{m}\left(\mathbf{E}_2\nabla\cdot\mathbf{P}_1 + \mathbf{E}_1\nabla\cdot\mathbf{P}_2\right) + \\ &-i\frac{e\mu_0}{m}\left(\omega_2\mathbf{P}_2\times\mathbf{H}_1 + \omega_1\mathbf{P}_1\times\mathbf{H}_2\right) + \\ &+\frac{\omega_1\omega_2}{en_0}(\mathbf{P}_2\nabla\cdot\mathbf{P}_1 + \mathbf{P}_2\cdot\nabla\mathbf{P}_1 + \mathbf{P}_1\nabla\cdot\mathbf{P}_2 + \mathbf{P}_2\cdot\nabla\mathbf{P}_2) \\ &-\frac{2}{3}\frac{\beta^2}{en_0}\left(\nabla\cdot\mathbf{P}_2\nabla\nabla\cdot\mathbf{P}_1 + \nabla\cdot\mathbf{P}_1\nabla\nabla\cdot\mathbf{P}_2\right) \end{aligned} \tag{S7a}$$

$$\begin{aligned}\mathbf{S}^{(3)}_{\omega_3} = &-\frac{\omega_1^2}{e^2n_0^2}\Big[\nabla\cdot\mathbf{P}_1(\mathbf{P}_1\nabla\cdot\mathbf{P}_1 + \mathbf{P}_1\cdot\nabla\mathbf{P}_1) + \\ &+\mathbf{P}_1\cdot\mathbf{P}_1\nabla\nabla\cdot\mathbf{P}_1\Big] + \frac{1}{27}\frac{\beta^2}{e^2{n_0}^2}\nabla(\nabla\cdot\mathbf{P}_1)^3\end{aligned} \tag{S7b}$$

describing cascaded and direct THG due to FE dynamics, respectively.

Simulation Methods and Results

Simulations for the linear responses in FTIR spectra

The absorption spectra of the InGaAs antennas array were presented in the main text measured with an FTIR setup. These absorption spectra could be well reproduced by numerical full-wave electromagnetic simulations carried out by the finite-element method package COMSOL Multiphysics v6.1. As shown in Fig. S1a, a three-dimensional model with Floquet periodic boundary conditions in both $x-$ and $y-$directions was built to capture the response of the antenna array, whose unit cell has the dimensions $P_x = 5\ \mu$m and $P_y = 2\ \mu$m. The antennas have near-trapezoidal $yz-$cross-sections (n-doped InGaAs and the extra-etched InP substrate), which were also considered in our numerical model. As indicated in Fig. S1a, we set the top and bottom bases of the InGaAs and InP parts to be 850 nm and 1350 nm, respectively. The heights of the two parts were 750 nm and 375 nm, respectively. The corners were rounded by the curvature of 100 nm. The trapezoidal cross-section was then extruded by the length of 2.2 μm to complete a three-dimensional geometry (geometry and mesh shown in Fig. S1a). The refractive indices of the InGaAs of different doping levels follow the Drude model: $\varepsilon_D = \varepsilon_\infty - \omega_\text{p}^2/(\omega^2 - i\omega\gamma_0)$, where the $\omega_\text{p} = 890.2,\ 818.7,$ and 678.3 THz for antennas with different doping levels, 10.2×10^{18} cm^{-3}, 8.6×10^{18} cm^{-3}, and 5.9×10^{18} cm^{-3}, respectively. The effective mass of the electrons is 0.041. The damping $\gamma_0 = 8.9$ THz, background permittivity $\varepsilon_\infty = 12$. The refractive index of InP is 3. It is worth noting that, to match the experimental linear responses (main text Fig. 1**d**), in the main text Fig. 1**e** we broaden the damping by 3 times (i.e., $3\gamma_0 = 26.7$ THz) due to the possible broadening after nanofabrication.

Since the experimental FTIR setup for absorption would generate a donut-shape incident light on the sample (numerical aperture = 0.3), our simulation could also mimic the realistic setup by applying an oblique incident plane wave with an angle ($\theta = 17.5°$) approximately. The experimental absorption was simulated as $1 - R - T$,

where the reflectance $R = |s_{11}|^2$ and the transmittance $T = |s_{21}|^2$. Since the co-polarization was utilized in FTIR, aligning to the antenna long axis, we could assume the incident wave vector k either in $xz-$ or $yz-$plane in the simulation. Since these two directions could form a full set of base vectors and arbitrary incident light could be decomposed onto such bases, our simulation should be able to grasp all the features in the experiments. Hence, in our 3D simulation presented in Fig. S1b, two distinct resonance peaks were observed in the spectra, consistent with the experimental FTIR spectra (main text) that exhibit doublet features. As the doping level decreases, the plasmonic resonances see a significant redshift along with the bulk plasma wavelengths. Single InGaAs antennas, instead of arrays, under normal incidence were also considered (Fig. S1c). The main resonances kept the same energy (Figs. S1b and c).

In addition, in Fig. S1d, we propose a 2D model that simulates the $xz-$cross section of the 3D antenna geometry for the purpose of validation. Given that the experimental setup employs light polarized along the long axis of the antenna, utilizing such a cross-section with x-polarized plane-wave excitation is expected to faithfully replicate the principal characteristics observed in the 3D scenario. Figure S1d, illustrates the normalized extinction of the corresponding 2D model. In this representation, the majority of features, e.g., resonances, were faithfully replicated, except for a blueshift of approximately 0.6 μm observed in the 2D model. This is due to the loss of the confinement of one dimension (y-dimension here), which could be understood by intuitive analogy to nanorods. It is anticipated that the resonances of the nanorods will exhibit a redshift when the aspect ratio is higher (e.g., length/depth $\sim$ 3 for the 3D antenna case, $\sim$ 0 for the 2D case). Overall, the 2D model effectively captures the information delivered by the 3D model in terms of linear response, though with acceptable blueshift. This validation is the first step that assures us of the credibility of the 2D implementation for hydrodynamics-driven nonlinear responses.

Hydrodynamic model for nonlinear responses

To explain the unconventional nonlinearity due to the free-electron dynamics, a hydrodynamic theory with Thomas-Fermi level approximation was applied.[3] From the experimental setup and the comparison of the linear-response spectra between the 3D and 2D model (Figs. S1**b**,**c** and **d**), we proved that the co-polarized light would mainly excite the plasmonic resonances supported by the xz-cross-section. Every plasmonic resonance in the 3D model could be captured by the 2D model except for a slight blueshift of about 0.6 μm. Combining the fact of computational capacity and the validity, we chose to use the 2D model to calculate the hydrodynamic nonlinear responses. It has to be emphasized that since we are exploring the surface effects that ask for delicate meshing elements, divergence brought by sharp corners of the antennas needs to be avoided by rounding curvatures. Curvatures with 0.12 μm radius were used to round the 2.2 μm $\times$ 0.75 μm InGaAs antenna (it reflects the xz-cross section of Fig. S1**a**). When comparing the results acquired by 2D simulations to the experiments, as shown in the main text Fig. 4, we could manually blueshift the pump wavelength by 0.6 μm for this 2D-3D compensation. Since all the linear plasmonic features are the same in 2D and 3D models (Figs. S1**c**,**d**), we believe this modification of the shifting still holds the validity for understanding the physics.

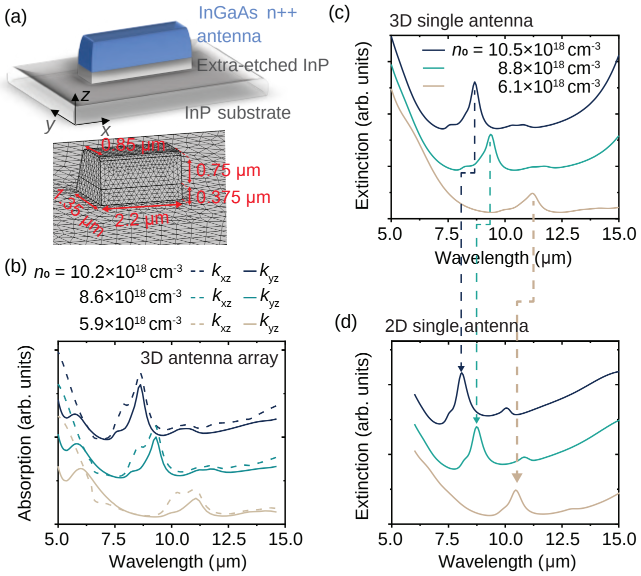

Fig. S1 Numerical simulations for the linear response: **a,** Geometry and mesh of the InGaAs antenna. The spectra of the structures with different doping levels under co-polarised excitation: **b,** the absorption of a 3D array, the extinctions of **c,** 3D single antenna , and **d,** 2D single antenna.

By considering the Thomas-Fermi kinetics and the hard-wall conditions, we could self-consistently solve the electromagnetic wave equations and hydrodynamic constitutive conditions together. Taking both the hydrodynamic direct and cascaded THG, and THG from lattice ($\chi^{(3)} = 1.4 \times 10^{18}$ m^2 V^{-2}) into account, we could calculate the power P of THG emission from a single antenna transmitted into the objective by integrating the Poynting vectors $\mathbf{S}_{\text{TH}}$ on the collection surface $\mathbf{A}$: $P = \int \mathbf{S}_{\text{TH}} \cdot \text{d}\mathbf{A}$. The numerical aperture of 0.5 was taken into account in the integration. It has to be noted that 3D simulation has one more dimension in the integration compared with the 2D simulation we are considering now. In the 2D simulation, rather than a surface integration over d$\mathbf{A}$, it is a line integration at the inner boundary of the perfectly matched layer. To match this difference in both unit and value, we can do one more modification. Since we are able to calculate the local-response model of a full 3D geometry without hydrodynamic terms, we could compare the value of the THG emission solely from lattice $\chi^{(3)}$ in 3D geometry with that from the reduced 2D geometry by norm.factor[m] $= \text{P}_{\text{3DTHG}}[\text{W}]/\text{P}_{\text{2DTHG}}[\text{W m}^{-1}]$. This normalized (correction) factor contains the information on the extra dimension (depth, y-axis) for collection and extra enhancement that 2D geometry does not have. After applying this factor to the nonlocal 2D model, we could also correct 2D hydrodynamic model to obtain the THG power.

When calculating the nonlinear coefficients η with a unit of cm^6 GW^{-3} in the main text Figs. 4 and 5, which is defined as the number of TH photons per fundamental-frequency input power cubic $\eta = N_{\text{THG}}/I_{FF}^3$, we need to normalize the TH power to the TH photon numbers with the relation $N_{\text{TH}} = P_{\text{TH}} \cdot t_{\text{pulse}}/\hbar\omega_{\text{TH}}$. Here, t_{pulse} is the

time duration of the pulse (between 360 fs to 430 fs measured in experiments), $\hbar\omega_{\mathrm{TH}}$ is the energy of a single TH photon related to the fundamental frequency given by the pump. After acquiring the number of TH photons, we could further normalize it by the pump I_{FF}^3 which ensures that this nonlinear coefficient η becomes pump-power independent because the THG follows a cubic power law. When comparing with, and explaining the real experimental nonlinear responses in main text Fig. 5, we could take two factors into account: i) the broadening (same as main text Fig. 1**e**), and ii) the number of antennas since there are more than one antenna excited by the laser. The full width at half maximum of the beam spot is estimated to be 80 μm, given by the periodicity, around 640 antennas were considered to contribute to the collected TH emission.

In addition, when comparing the TH nonlinear coefficients under three specific situations: **(a)** conventional local-response theory with only lattice $\chi^{(3)}$ (main text, Figs. 4**b, c**); **(b)** hydrodynamic theory with both hydrodynamic terms and lattice $\chi^{(3)}$ (main text, Figs. 4**c, d**); **(c)** the reference with only hydrodynamic terms without $\chi^{(3)}$ (SI, Fig. S2), we could simply switch on and off the corresponding nonlinear polarizations for controlling the sources. From Fig. S2 we could find that without the lattice $\chi^{(3)}$ as a baseline, the minima of the nonlinear coefficient occurred in the dielectric regime is approaching 10^{-3} which is far below that in the other situations. The fact that the nonlinear coefficient is not strictly zero and there are some very weak blueshifting fringes on doping in the dielectric regime is possibly due to the longitudinal bulk plasmons wave allowed by the hydrodynamic theory with optical nonlocality. Bulk plasmons are weak and usually negligible compared with the surface plasmons utilized in this article.

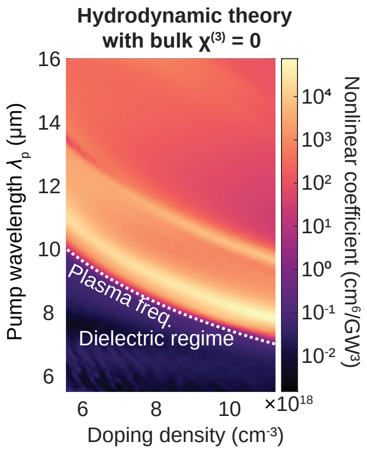

Fig. S2 THG with only hydrodynamic nonlinear sources: Map of the nonlinear coefficient on different pumping wavelengths and doping density based on solely the hydrodynamic nonlinear sources (lattice contribution $\chi^{(3)} = 0$).

To further understand the THG dependence on the doping density, we directly calculated the nonlinear coefficients from the 2D equivalent model without taking the 3D normalization (correction) factor into account (Fig. S3) to avoid ambiguity. Notably, in the main text Fig. 5, we calculated the correction factors (see the methods above) of four differently doped samples ($\lambda_{\mathrm{p}} = 9.62, 7.97, 7.33\ \mu$m, and undoped) against continuous fundamental field λ_{FF}. However, here, we need a different set of normalization factors that would give a correction according to the continuous doping density. It will arouse further troubles since they are two sets of different correction factors based on different peaks. Yet, we want to stress that, even without the dimensional correction, the trends of the curves and specific scatters in Fig. S3 give qualitatively good reproduction of all the results in Figs. 5a,b. Now, the unit of the nonlinear coefficient of the 2D system becomes (cm^6 GW^{-3} μm^{-1}) rather than (cm^6 GW^{-3}) of the 3D system, since we lost one dimension. The continuous curves in Fig. S3 clearly explain the differences between hydrodynamic and local-response theory. While the λ_{FF} is below plasmon frequency (i.e., in the plasmonic-resonance or metallic regimes in Fig. S3a), the free-electrons nonlinearity will play a dominant role over the bulk $\chi^{(3)}$ predicted by the local-response theory (Fig. S3b).

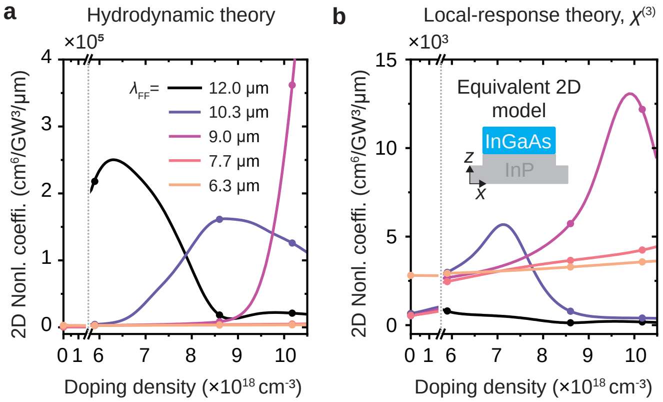

Fig. S3 THG with equivalent 2D model against doping density: Calculated THG from the equivalent 2D model without 3D-correction based on the hydrodynamic **a** or local-response theory **b**. The dots correspond to the specific doping levels used in the experiment. The trend of the curves qualitatively explains Figs.4a-c in the main text.).

Third harmonic emission from InGaAs thin films

The hydrodynamic theory describes the electron nonlinearities in doped thin films as well as in nanoantennas. We show in this section the experimental results of the TH experiments on thin films. Figure S4 shows a THG map of the InGaAs thin film on the InP substrate. The THG emission comes almost entirely from the thin film, while the substrate does not contribute significantly.

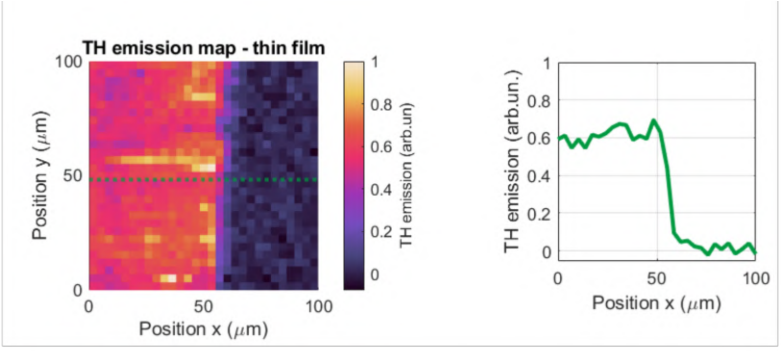

Fig. S4 (a) THG map of the InGaAs thin layer on InP substrate. (b) Horizontal cut of the THG map that provides an estimation of the spatial resolution of the measurement. The wavelength $\lambda_{mathrmFF}$ = 9 μm.

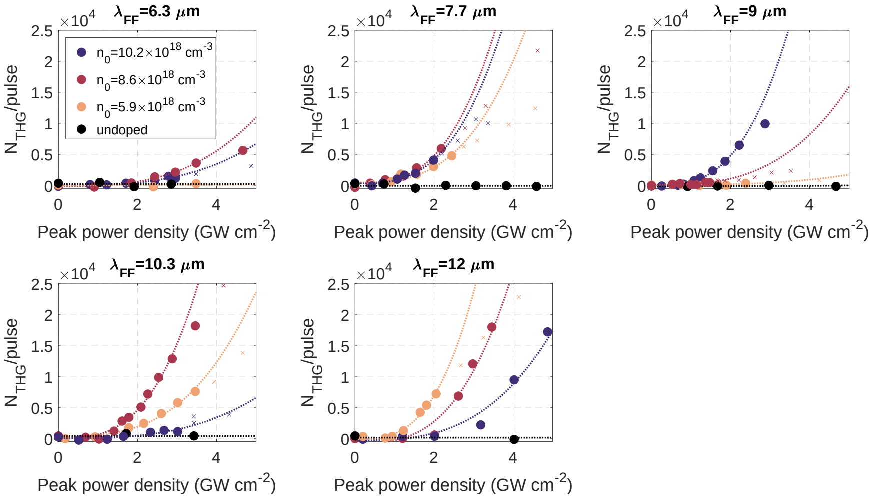

Fig. S5 Mid-infrared THG from thin films: peak power density dependence of the THG at different doping levels (color coded) and different fundamental field wavelengths λ_{FF} (different panels).

Figure S5 shows the peak power density dependence of the TH emission for four different fundamental field wavelengths. From the peak power density dependence we extracted the TH efficiency η_{exp} for each wavelength.

Figure S6 shows the comparison of experimental TH efficiency and the results of the numerical calculations. The local response model and the hydrodynamic theory predict different quantitative behavior but a similar qualitative behavior. Therefore, the comparison with the experimental data is not clear enough to distinguish from the two contributions. In order to enhance the effects of hydrodynamic nonlinearity and its differences with respect to the local-response theory, we have performed the experiment on nanostructured antennas, as presented in the main text.

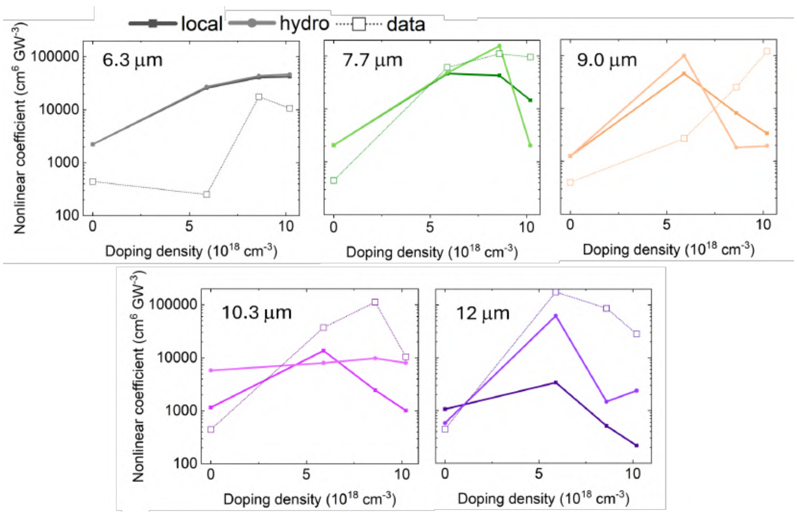

Fig. S6 Mid-infrared THG from thin films. Nonlinear TH coefficient as function of doping levels for different fundamental field wavelengths λ_{FF}. All the experimental nonlinear efficiencies have been multiplied by 200 to better visualize their qualitative behavior with respect to the numerical calculations.

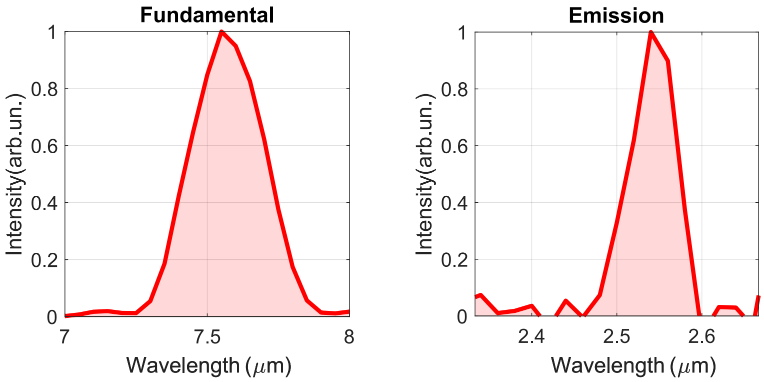

Fig. S7 Third harmonic emission spectrum: example of acquired spectra for the exciting pump beam (left) and for the respective third harmonic emission (right)

Third harmonic emission spectra

Figure S7 shows the spectra of the fundamental optical beam and its third harmonic emission measured with the monochromator as described in the Methods section. This observation confirms that we measure third harmonic emission and that we can filter efficiently the third harmonic signal from the fundamental field.

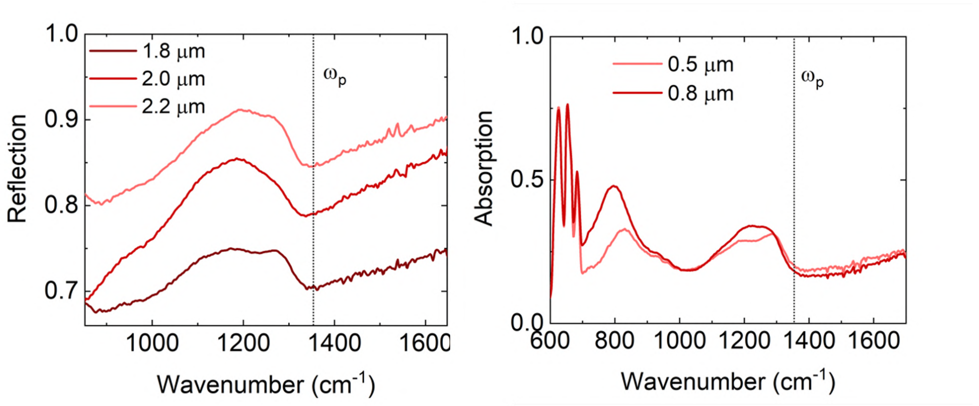

Fig. S8 Left: localized plasmonic resonance of the nanoantennas for three different antenna lengths (1.8 μm, 2.0 μm, and 2.2 μm). The resonance peak does not shift significantly by changing the antenna length. b) Absorption spectra of the same antennas but with orthogonal linear polarization (parallel to the short axis of the antenna): one can observe two plasmonic resonances (substrate-semiconductor mode and semiconductor-air mode) not shifting in frequency even though the antenna width changes dramatically from 0.8 to 0.5 μm.

Varying the antenna lengths: plasmonic resonances and THG

Figure S8 shows the infrared spectra of antenna arrays with different antenna dimensions. The main observation is that the high-frequency plasmonic resonances do not shift significantly by changing the antenna dimensions since the resonances are higher-order modes which are close to the plasma wavelength of the InGaAs layer. The low-energy resonance along the long axis was not observed in the spectra because the energy lay in the range covered by the phonon noises (below 700 cm^{-1}). Notably, we do observe the low-energy resonances when the polarization is along the short axis of the antennas. In this case, since the resonance is far from the plasma wavelength, we do observe an energy shift when the antenna short axis changes from 0.5 μm to 0.8 μm.

Figure S9 shows the third harmonic generation from antenna arrays of different dimensions. Specifically, the long axis of the antenna changes from 1.8 μm to 2.2 μm.

As it can be observed from the inset of Figure S9, the TH efficiency does not depend significantly from the antenna length since their plasmonic resonance does not shift with the length but with the plasma wavelength of the InGaAs layer.

Mid-infrared spot size for THG

The minimum available spot size is close to the diffraction limit when focusing with the Cassegrain objective, as directly measured by us with the knife-edge method (see Figure S10). However, the nonlinear emission experiment is performed slightly out of focus with an approximately circular illumination area on the sample of 80 μm encompassing a larger portion of the antenna array. This measurement configuration

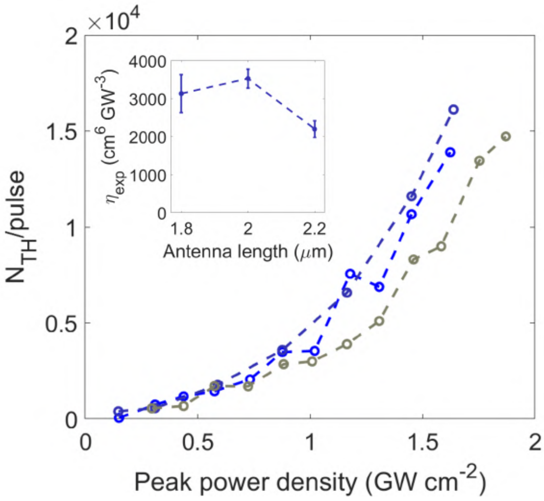

Fig. S9 TH signal as a function of peak power density for three different antenna lengths, i.e. 1.8 μm, 2.0 μm, and 2.2 μm. The inset shows the extracted TH efficiency.

has several advantages. Firstly, non-negligible emission from the substrate occurs at the focus and the experiment was consequently performed at a position where the contrast between emission from the antennas and from the substrate was maximized. Moreover, moving away from the focus we increase the available range of incoming peak power densities where the antennas are driven in a purely perturbative regime (before the onset of saturation effects), while at the same time taking advantage of volume effects since we integrate the emission from many antennas.

Nonlinear active volume for THG

Figure S11 clarify the crucial role of the active volume that contributes to the THG according to the hydrodynamic theory. The nonlinear response is determined by terms that include gradients of the polarization (Eq. 2b of the main text). In a metal, the non-equilibrium carrier density induced by the electromagnetic wave has a sharp gradient of polarization at the interface and a vanishing gradient elsewhere. Consequently, the portion of the material that effectively contributes to the nonlinearity is very small. In contrast, in doped semiconductors, the induced charge density spread more into the bulk, leading to an extended active volume (see Figure S11). As a result, the nonlinear optical response is significantly enhanced in semiconductors.

THG data in linear scale

Figure S12 shows the same data shown in Figure 4 of the main text but in linear scale. The linear scale makes clear the quantitative difference between hydrodynamic and local-response predictions compared to the experimental data. It is essential to note that the most evident difference between the models occurs in the metallic regime (high doping density, when the plasma wavelength is shorter than the FF wavelength).

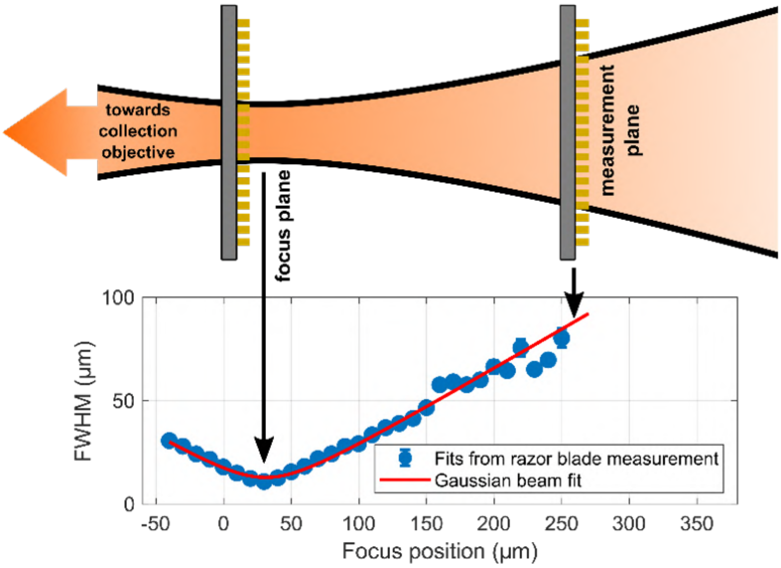

Fig. S10 Sketch of the position of the sample used for the experiment and spot size as measured with the knife-edge method.

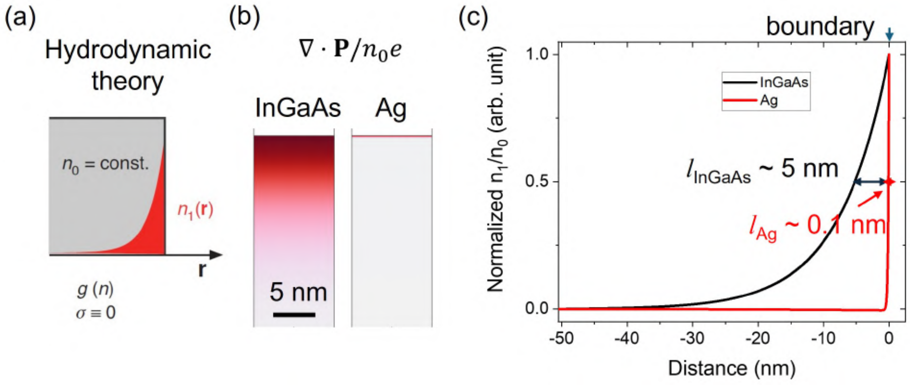

Fig. S11 **Active volumes for optical nonlinearity.** (a) n_0 is the equilibrium charge density and n_1 is the photo-induced non-equilibrium charge density. Adapted from *Ciracì et al.* [5] (b) Charge density distribution close to the surface of InGaAs and silver layers. (c) Plot of the charge density as a function of depth into the material. The charge density spread much more into the bulk of the semiconductor, making much larger the volume of the material that contributes to THG.

In the dielectric regime (right panel), there is no difference between the models since the free electrons do not contribute to the nonlinearity.

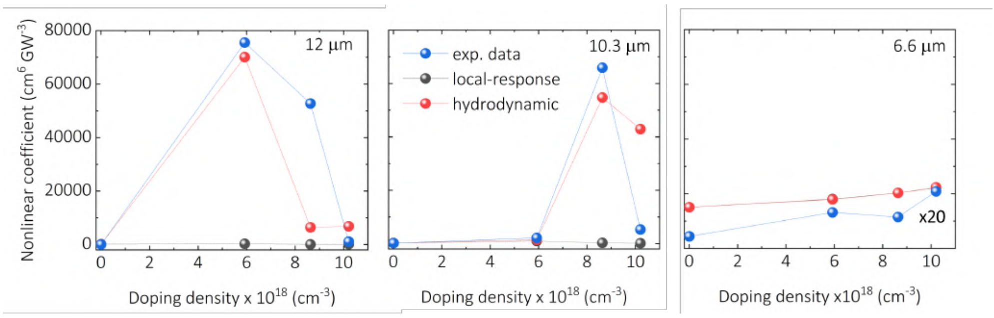

Fig. S12 Theory-experiment comparison. Plot in linear scale of the nonlinear coefficient where the experimental data agrees better with the hydrodynamic model for third harmonic generation.

References

[1] C. Ciracì, F. Della Sala, Quantum hydrodynamic theory for plasmonics: Impact of the electron density tail. Physical Review B **93**(20), 205405 (2016). https://doi.org/10.1103/physrevb.93.205405

[2] C. Ciracì, Current-dependent potential for nonlocal absorption in quantum hydrodynamic theory. Physical Review B **95**(24), 245434 (2017). https://doi.org/10.1103/physrevb.95.245434

[3] F. De Luca, M. Ortolani, C. Ciracì, Free electron nonlinearities in heavily doped semiconductors plasmonics. Physical Review B **103**(11), 115305 (2021). https://doi.org/10.1103/physrevb.103.115305

[4] G. Manfredi, How to model quantum plasmas. arXiv (2005). https://doi.org/10.48550/arxiv.quant-ph/0505004. quant-ph/0505004

[5] C. Ciracì, R. Jurga, M. Khalid, F.D. Sala, Plasmonic quantum effects on single-emitter strong coupling. Nanophotonics **8**(10), 1821–1833 (2019). https://doi.org/10.1515/nanoph-2019-0199. URL https://www.editorialmanager.com/nanoph/auth_ndauthApprove.asp?SessionThreadIdField=a4a1be0f-ad3c-4edb-a534-fa0c5338976a
